# Computational Simulation of Exosome Transport in Tumor Microenvironment

**DOI:** 10.3389/fmed.2021.643793

**Published:** 2021-04-13

**Authors:** Roy Koomullil, Behnam Tehrani, Kayla Goliwas, Yong Wang, Selvarangan Ponnazhagan, Joel Berry, Jessy Deshane

**Affiliations:** ^1^Department of Mechanical Engineering, University of Alabama at Birmingham, Birmingham, AL, United States; ^2^Department of Medicine, Division of Pulmonary Allergy and Critical Care Medicine, University of Alabama at Birmingham, Birmingham, AL, United States; ^3^Department of Pathology, University of Alabama at Birmingham, Birmingham, AL, United States; ^4^Department of Biomedical Engineering, University of Alabama at Birmingham, Birmingham, AL, United States

**Keywords:** exosome, interstitial fluid flow, diffusion, computational modeling, transport equations

## Abstract

Cellular exosome-mediated crosstalk in tumor microenvironment (TME) is a critical component of anti-tumor immune responses. In addition to particle size, exosome transport and uptake by target cells is influenced by physical and physiological factors, including interstitial fluid pressure, and exosome concentration. These variables differ under both normal and pathological conditions, including cancer. The transport of exosomes in TME is governed by interstitial flow and diffusion. Based on these determinants, mathematical models were adapted to simulate the transport of exosomes in the TME with specified exosome release rates from the tumor cells. In this study, the significance of spatial relationship in exosome-mediated intercellular communication was established by treating their movement in the TME as a continuum using a transport equation, with advection due to interstitial flow and diffusion due to concentration gradients. To quantify the rate of release of exosomes by biomechanical forces acting on the tumor cells, we used a transwell platform with confluent triple negative breast cancer cells 4T1.2 seeded in BioFlex plates exposed to an oscillatory force. Exosome release rates were quantified from 4T1.2 cells seeded at the bottom of the well following the application of either no force or an oscillatory force, and these rates were used to model exosome transport in the transwell. The simulations predicted that a larger number of exosomes reached the membrane of the transwell for 4T1.2 cells exposed to the oscillatory force when compared to controls. Additionally, we simulated the interstitial fluid flow and exosome transport in a 2-dimensional TME with macrophages, T cells, and mixtures of these two populations at two different stages of a tumor growth. Computational simulations were carried out using the commercial computational simulation package, ANSYS/Fluent. The results of this study indicated higher exosome concentrations and larger interstitial fluid pressure at the later stages of the tumor growth. Quantifying the release of exosomes by cancer cells, their transport through the TME, and their concentration in TME will afford a deeper understanding of the mechanisms of these interactions and aid in deriving predictive models for therapeutic intervention.

## Introduction

The tumor microenvironment (TME), which contains multiple cell types, blood and lymph vessels, and extracellular matrix (ECM), is often immunosuppressive, blocking anti-tumor immunity, and mediating drug resistance (1). Previous studies have shown that tumors have significantly increased hydrostatic pressure and solid force (tension, compression, and shear), and these forces activate signaling pathways and drive changes in tumor cell proliferation, adhesion, migration, and survival (2). Emerging evidence indicates that biomechanical forces in the TME may influence tumor progression due to anti-tumor immune response through cancer cell-immune cell crosstalk, including that mediated by vesicular transport (3, 4). As a key mediator of cell-cell signaling within the TME, tumor cell exosomes have emerged as important regulators of several aspects of tumor progression (5), including tumor initiation, progression and invasion, and preparation of pre-metastatic niches (6, 7). Exosomes are 50–150 nm membrane-bound extracellular vesicles (EVs) released by various cells in the TME, including tumor cells (8). Exosomes contain proteins and microRNAs that can impact immune cell function. We and others have demonstrated that tumor-derived exosomes promote macrophages polarization (9–11). They contribute to immune regulation and can create an immuno-privileged environment within tumors (8, 12).

Exosomes secreted by the primary tumor lesion have been shown to aid in the formation of metastatic lesion in distant tissues (i.e., pre-metastatic niche formation) before the cancer cells themselves migrate to the particular tissue. In 2012, Peinado et al. (13) showed that when exosomes derived from melanoma cells, with highly metastatic potential to the lungs, were administered to mice, they promoted vascular permeability through the introduction of bone marrow cells to the lungs and thereby contributed to the formation of a pre-metastatic niche. Similarly, pancreatic cancer cell-derived exosomes formed a pre-metastatic niche for liver metastasis (6, 14). Internalization of pancreatic cancer cell-derived exosomes containing macrophage migration inhibitory factor by hepatic Kuppfer cells, induced fibronectin and TGF-β production by hepatic stellate cells and ultimately formed a pre-metastatic niche through introduction of bone marrow cells to the liver (15). In the context of infections, exosome transfer by lymphatic flow from the periphery to the lymph node has been proposed to be a mechanism for rapid exchange of infection-specific information that precedes the arrival of migrating cells, thus priming the node for a more effective immune response (16). Recent studies have also investigated the utility of exosomes for drug delivery and as carriers of bioactive molecules (17, 18). *In vivo* studies demonstrate that after being internalized at the administration site and transferred to the systemic circulation, exosomes pass blood–tissue barriers and arrive in each tissue (17–19). Intravenously administered exosomes have been shown to be mainly distributed in the organs with a mononuclear phagocyte system such as the liver, spleen, lungs, and kidneys (6, 13, 20). In our own studies, intratumoral injection of tumor-derived exosomes resulted in internalization by mononuclear phagocytes including immune suppressive myeloid-derived suppressor cells (21). The release of exosomes by cancer cells, their transport through the TME, and their crosstalk with immune cells once they reach the tissue are not well-understood. Even though the importance of exosomes in TME is well-documented in literature, tools to quantify exosomes in TME are lacking. This necessitates the development of a numerical approach to simulate the transport of exosomes and estimate concentration gradients of exosomes in TME.

With the advent of faster and better computational resources and algorithms, researchers have modeled various aspects of the TME including simulation of tumor growth in the microenvironment (22–25). Several studies have comprehensively reviewed the existing models for tumor growth, tumor progression and morphology (22, 23). These models took into account the genetic characteristics of the tumor and the TME. Bresch et al. (24) used partial differential equations to model tumor growth and to estimate tumor densities, and level set methods to model the membrane. Sciumè et al. (25) developed an approach to model the TME with tumor and healthy cells, interstitial fluid, and extracellular matrix using continuum mechanics principles. Crespo et al. documented the importance of mathematical modeling of heterogeneous systems in tumor microenvironments and interplay of these elements in TME (26). Further, they elucidated the use of computational simulations in personalized cancer therapy and precision medicine.

Various studies have addressed the computational simulation of interstitial fluid flow and estimation of shear stress and fluid pressure (27–30). Mitchell and King (27) recognized that mechanical forces such as fluid shear stress can influence cancer metastasis. They used the Stokes equation, Darcy's law, and Brinkman equation to estimate the fluid properties in TME. Welter and Rieger (29) postulated that the elevated interstitial fluid pressure due to a tumor could influence the interstitial fluid flow and delivery of drugs and nutrients to the cells. They modeled TME as a porous medium and the movement of chemicals in TME was modeled using a transport equation. A non-invasive technique to estimate the response of cancer treatment using imaging, pharmacokinetics, and interstitial fluid flow modeling was presented by Swinburne et al. (30). In this modeling, the governing equations were taken as the Navier-Stokes equation and the extracellular matrix was modeled as a porous medium. Voronov et al. (31) used a numerical approach to estimate the fluid shear stress in a scaffold. A high resolution micro-CT was used to extract the detailed geometry of the scaffold and the lattice Boltzmann method used for the modeling of fluid flow inside the scaffold. Kim et al. used a medical imaging technique (32) to extract high fidelity 3-D geometry of a tumor and mathematical models to delineate blood flow and molecular transport. The goal of this multi-scale modeling was to develop a predictive model for tumor angiogenesis. Another approach to model TME are cell or agent-based methods, which simulate the dynamic evolution of individual entities called agents based on a set of rules (33–36). A survey of different cell-based mathematical models to analyze TME and their relative strengths and weaknesses are presented in Rejniak and McCawley (37). Cell-based approaches are particularly useful in modeling individual cell behavior such as polarization of immune cells, which provide insight into the dynamic processes in heterogeneous TME.

Although various aspects of TME have been modeled using computational simulations, the computational tools to simulate exosome transport in TME are lacking. Quantifying exosome concentration and factors that regulate their interactions with various cells in the TME will afford a deeper understanding of the mechanisms of interaction and aid in deriving predictive models for therapeutic intervention. This article focuses on a computational technique to model the transport of exosomes in the TME by taking into account exosome production by cancer cells, exosome advection due to interstitial flow, and exosome diffusion due to concentration gradients.

## Materials and Methods

### Computational Modeling

In this study, because of the sub-micron size of exosomes, their movement in the TME was treated as a continuum and modeled using an advection-diffusion transport equation, with advection due to interstitial flow and diffusion due to concentration gradients (38). In general, an exosome transport equation can be written as,

(1) $$\begin{array}{l} \frac{\partial c_{k}}{\partial t}+\frac{\partial u_{i}c_{k}}{\partial x_{i}}=\frac{\partial }{\partial x_{i}}\left(\Gamma_{k}\frac{\partial c_{k}}{\partial x_{i}}\right)+S_{c_{k}},\;k=1,\ldots ,N \end{array}$$

Where *c*_*k*_ is the concentration of the *k*^th^ type of exosome in a heterogeneous field with *N* number of exosome types, *u*_*i*_ is the interstitial fluid velocity components, Γ_*k*_ is the diffusion coefficient, and *S*_*ck*_ is the exosome source or sink term. In the above equation, the second term on the left-hand side models the movement of exosomes due to interstitial fluid flow, the first term on the right-hand side models the diffusion of exosomes due to concentration gradients, and the last term on the right-hand side represents the production of exosomes by donor cells (tumor cells) and uptake of exosomes by receptor cells (immune cells). In this study, the uptake of exosomes by immune cells were not taken into consideration. The diffusion coefficient for the simulation was taken from the data published in the literature (38–42). The interstitial fluid flow in TME is governed by the Navier-Stokes equations (43–45), which is the mathematical representation of the conservation of mass, momentum, and energy. The fluid flow in TME is incompressible in nature, and heat transfer is also not an important factor for the flow in TME (46). Therefore, the energy equation was not considered for the following simulations. The turbulent effects were not modeled due to the fact that the Reynolds number for the interstitial flow was very small. Therefore, the laminar flow assumption was used for the simulations.

Computational simulations presented in this article were carried out using the commercial computational simulation package called ANSYS/Fluent (47). It has capabilities to model Navier-Stokes and transport equations for different flow regimes using various numerical schemes, and is commonly used in academia and industries. In the present simulations, the transport of exosome in TME was carried out in two stages using a staggered approach. In the first stage the Navier-Stokes equations were solved to estimate the velocity field. In the second stage, the transport equations were solved using the velocity field predicted from the first step. In these simulations, it was assumed that the transport of exosomes in TME would not affect the underlying velocity field. Geometries for TME, presented in this article were extracted from the images created using Biorender (48). These images were segmented using edge detection techniques to get the outer boundaries of tumor and immune cells present in TME, with the application of the geometry modeling program SpaceClaim available in Ansys (47).

### Validation of the Numerical Approach

Validation of the numerical methods using experimental data or analytical solution of an appropriate problem is an important step in computational simulations. The numerical approach to solve the advection-diffusion equation used in this study was validated using a Gaussian source in a planar shear flow (49, 50). The computational domain for this simulation was taken as a square with dimension 100 × 100 mm, with the origin at the geometric center. In this test case, the *y*-component of the velocity was taken as zero and the *x*-component of the velocity was assumed to vary linearly as *U* + λ*y*, where *U* and λ are constants. At time equals zero, the concentration of the transport variable was taken as an impulse function at the origin, multiplied by a constant *M*. The exact solution of the concentration for this Gaussian source in a planar shear flow at any spatial location and at any time is given by the relation (49, 50),

(2) $$\begin{array}{l} c\left(x,y,t\right)=\\ \frac{M}{4\pi Dt{\left(1+\frac{{\left(\lambda t\right)}^{2}}{12}\right)}^{\frac{1}{2}}}\;\exp\left\{-\frac{1}{4Dt}\left[y^{2}+\frac{{\left(x-\left(U-0.5\lambda y\right)t\right)}^{2}}{\left(1+\frac{{\left(\lambda t\right)}^{2}}{12}\right)}\right]\right\} \end{array}$$

Where *c* is the concentration and *D* is the diffusion coefficient. More details on the analytical solution of this benchmark test case can be found in references (42, 43). Numerical values of different parameters used in this benchmark test case are listed in Table 1. To keep the concentration as finite values in the computational domain at the beginning of the simulation, the exact solution at time equals 1 min was used.

**Table 1:** Parameters used for the benchmark test case.

**Variable**	***U***	***λ***	***M***	***D***
Numerical value	5.0 × 10^−6^ (m/s)	5.0 × 10^−4^ (1/s)	1.0	1.0 × 10^−8^ m^2^/s

### Cell Culture and Exposure to Strain

**Figure 1:**
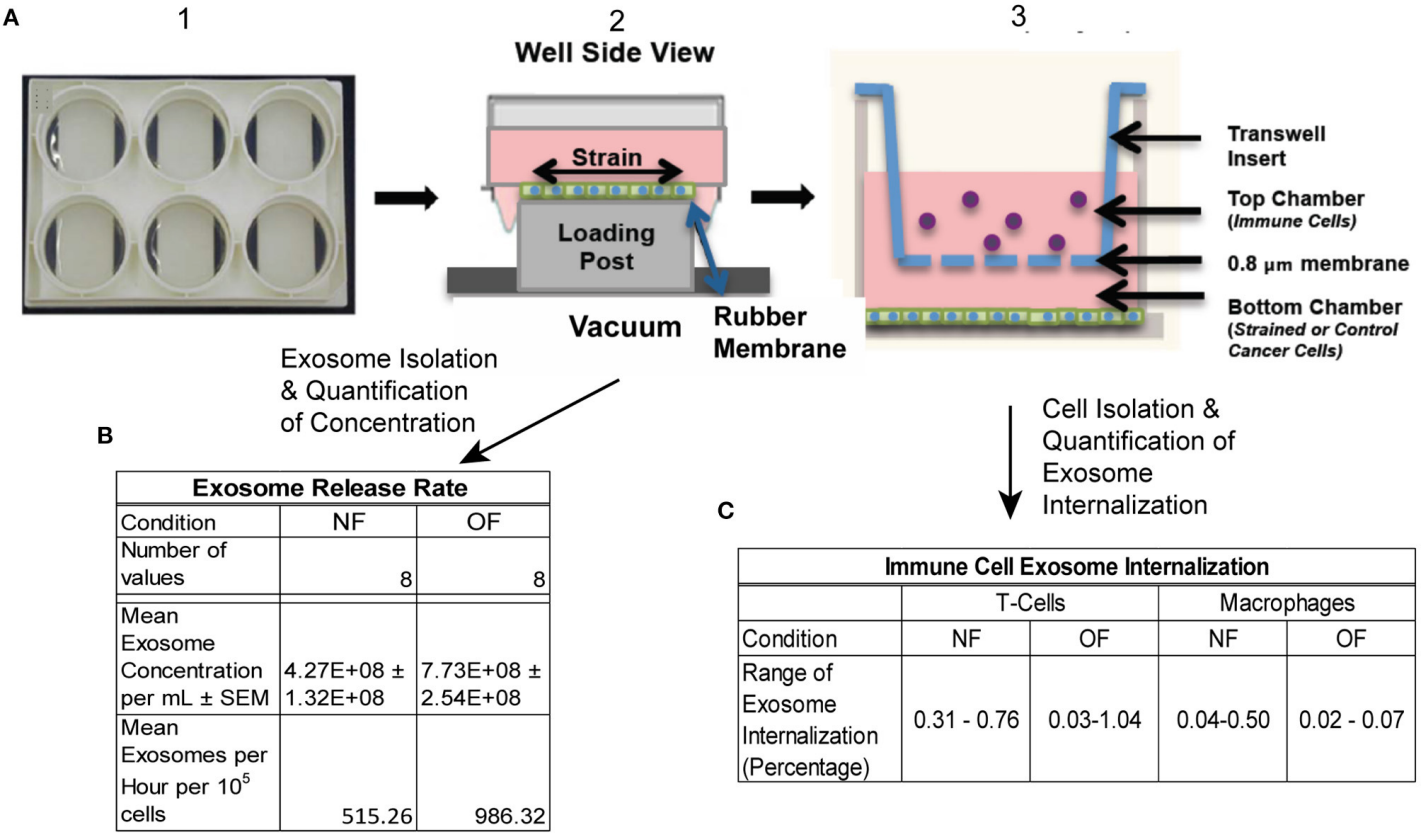
Schematic of experimental protocol. **(A)** 4T1.2 cells were seeded in BioFlex plates (1), once cells reached confluence they were exposed to OF strain (or NF) (2). Strain exposed (or NF) cells were either put into co-cultured with immune cells (T cells and macrophages) using transwell (3) or conditioned media was collected for exosome isolation. **(B)** Exosome release rate quantified following OF or NF. *n* = 8 per condition from 3 experiments, 2–3 replicates per experiment. **(C)** Exosome internalization by T cells and macrophages was quantified using Imagestream flow cytometry, *n* = 2–3 per condition.

The rate of release of exosomes is influenced by biomechanical forces acting on the tumor cells, and to quantify this, an experimental setup depicted in Figure 1 was used. The murine TNBC cell line 4T1.2 (an aggressive clone derived from 4T1) was obtained from Dr. Robin L. Anderson's laboratory (Peter McCallum Cancer Center, Australia). 4T1.2 cells were cultured in Dulbecco's Modified Eagle Medium (DMEM) supplemented with 10% FBS and 10 mM HEPES (MP Biomedicals, Santa Ana, CA). Prior to the exposure to tensile strain, the 4T1.2 cells were stained with the lypophilic dye PKH26 Red Fluorescent Cell Linker (Sigma, St. Louis, MO), per the manufacturer's instructions. 2.5 × 10^5^ 4T1.2 were seeded on collagen coated 6 well UniFlex culture plates (Flexcell International Corporation, Burlington, NC) and cultured to confluence. Once confluent, the media was changed to exosome depleted growth media and the plates were subjected to 10% uniaxial oscillatory strain at 0.3 Hz for 48 h, 10% constant strain for 48 h, or no strain for 48 h using a FlexCell FX-6000 or FX-5000 Tension System.

### Preparation of Exosome-Depleted Media

The exosome-depleted medium was prepared as previously described (51). Briefly, DMEM media supplemented with 20% FBS was centrifuged using an ultracentrifuge overnight at 100,000 × g at 4°C. The supernatant was filtered through a 0.2 μm cellulose acetate filter (Corning, NY). The exosome depleted media was then diluted 1:1 with DMEM to make a final concentration of 10% FBS and 10 mM HEPES.

### Isolation of Exosomes From Conditioned Media and Quantification of Concentration

Extracellular vesicles (EVs) were isolated as previously described (21). Briefly, conditioned media was collected following 48 h exposure to NF or OF and centrifuge at 2,000 × g to remove any cell fragments or apoptotic bodies. The supernatant was then incubated with the Total Exosome Isolation Reagent for Cell Culture Media kit (ThermoFisher, Waltham, MA) per the manufacturer's protocol. Purified exosomes were stored in 50 μl of PBS at −80°C. Exosome concentration was quantified using Imagestream flow cytometry.

### Immune Cell Exosome Internalization

0.4 μm pore size transwell filters (MilliporeSigma, Burlington, MA) were added on top of strained or control 6 well plates containing PKH26^+^ 4T1.2 cells. Subsequently, 5 × 10^4^ purified naïve lung macrophages (CD11b^+^CD64^+^), or 3 × 10^5^ purified splenic T cells (CD45^+^CD8^+^), or a mixture of these cell populations (1:3 ratio of macrophages to T cells, total of 2 × 10^5^) were added into the top well of the transwell filter. After 24 h, cells on the upper surface of the transwell filter were removed and tumor cell exosome internalization was evaluated using ImageStream flow cytometry, evaluating cell specific markers CD8 and CD11b, along with PKH26, to indicate exosome internalization.

## Results

### Results From the Validation of the Numerical Approach

**Figure 2:**
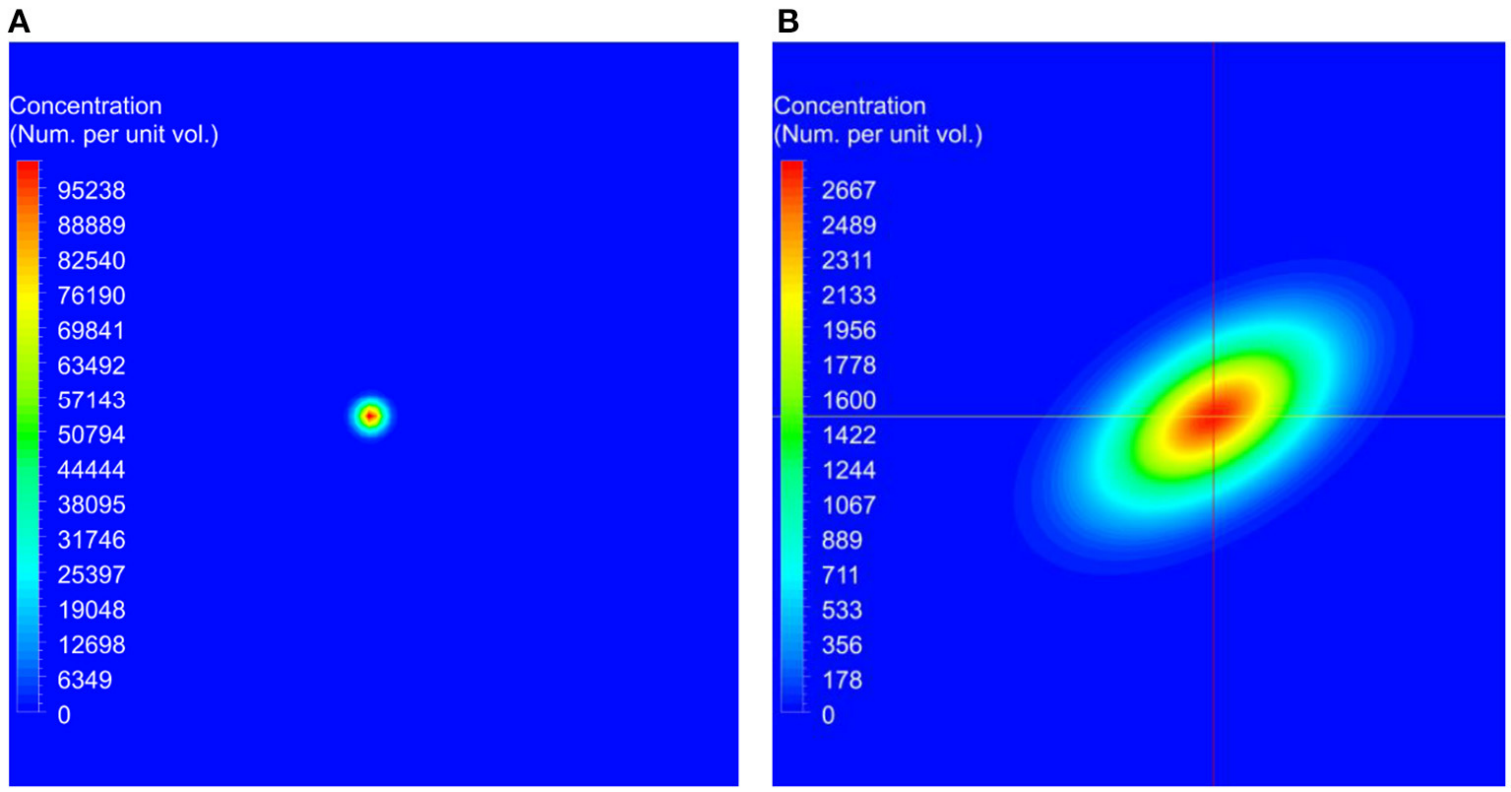
The initial conditions used for the benchmark simulations and the predicted concentration at the end of the simulation. In these figures, red represents higher concentrations and blue represents lower concentrations. **(A)** Initial concentration used at the beginning of the simulation. **(B)** Computed concentration after 45 min.

**Figure 3:**
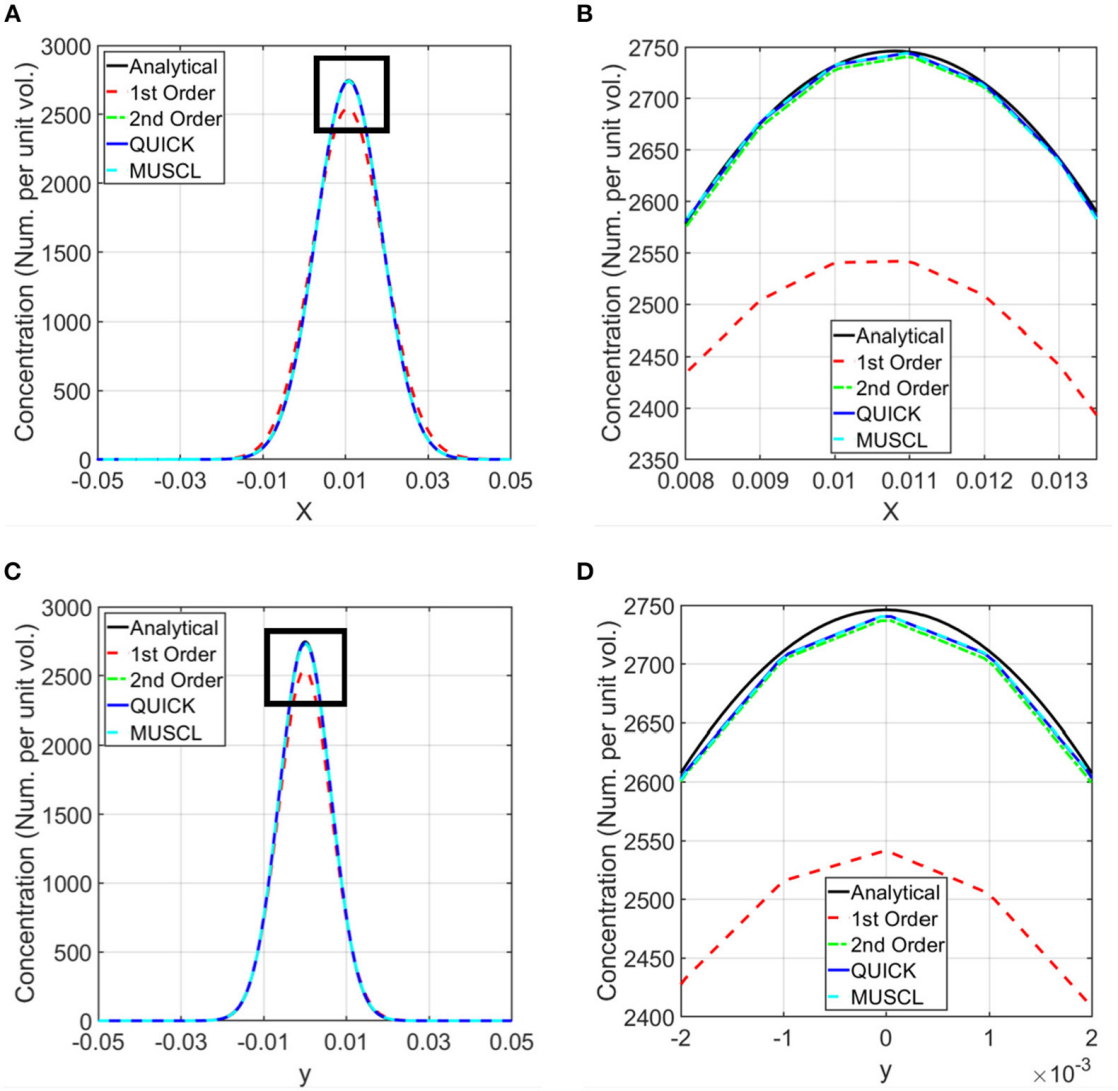
Comparison of computed concentrations using different numerical schemes with the analytical solutions. Simulations were conducted using first order, second order upwind, second order QUICK, and third order MUSCL schemes for the discretization of the spatial derivatives in the transport equation (37–39). Computed concentrations are compared with the analytical results along the horizontal and vertical lines shown in Figure 2B. **(A)** Comparison of the concentration along the horizontal line. **(B)** Zoomed in view of the comparison in the rectangle marked in **(A)**. **(C)** Comparison of the concentration along the vertical line. **(D)** Zoomed in view of the concentration in the rectangle marked in **(C)**.

For the simulation of the validation test case, the concentration of the transport variable at the beginning of the simulation is shown in Figure 2A, and the concentration after 45 min is shown in Figure 2B. These data show the advection due to the shear flow and diffusion due to the concentration gradient. Also, it can be seen from the result that as time progresses, the concentration gets distorted due to the velocity gradient normal to the flow direction. Computations were carried out to calculate the concentrations at 45 min using different numerical schemes and a quantitative comparison of the computed results with the analytical data is presented in Figure 3.

### 3-D Transwell Modeling

**Figure 4:**
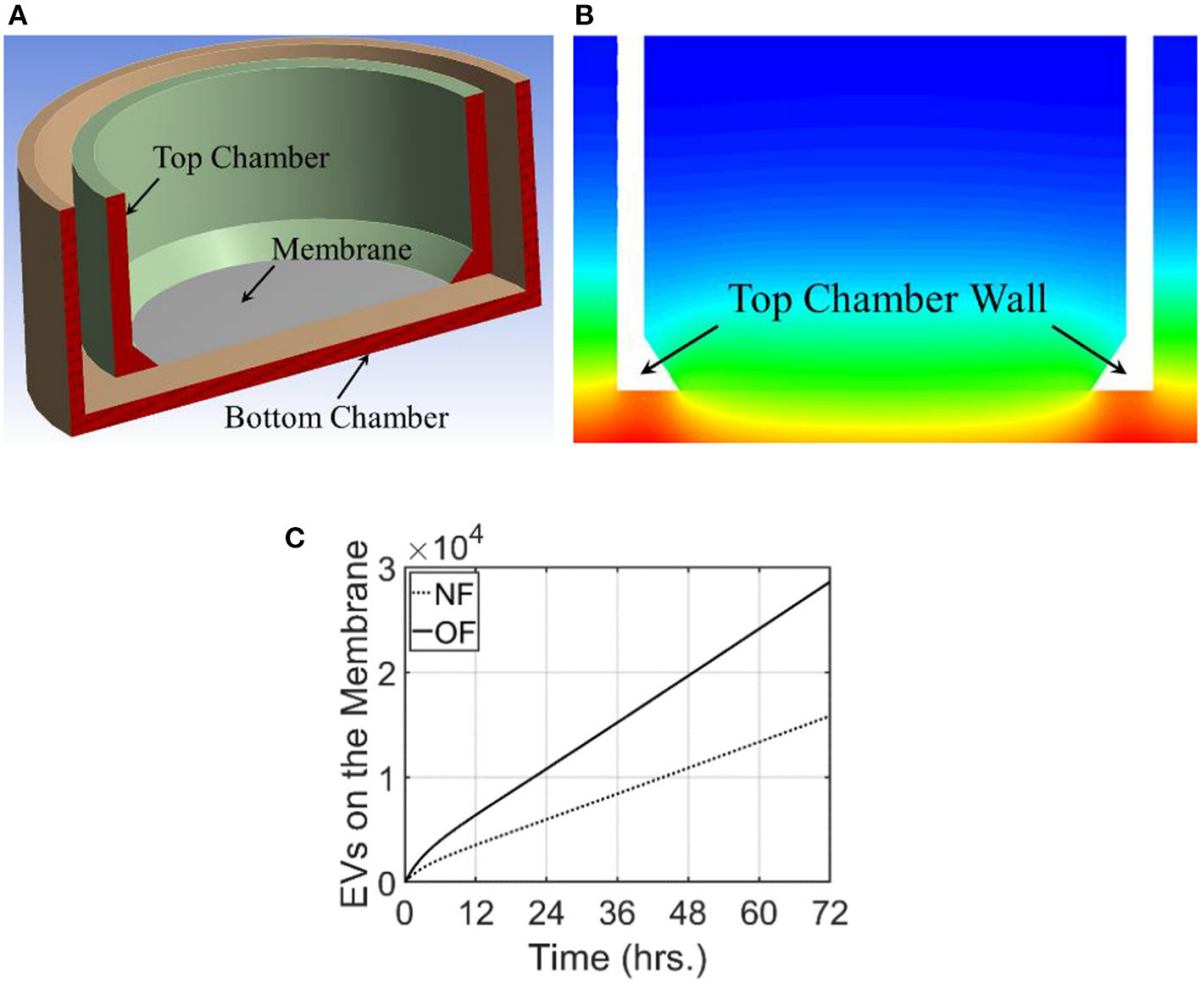
Computational domain and the results from modeling of exosome transport in a transwell. For this modeling, exosome release rate for the cells placed at the bottom surface is taken as 515.3 and 986.3/h./10^5^ cells for NF and OF, respectively. **(A)** A cross-sectional view of the 3-dimensional computational domain used for the simulation. **(B)** Exosome gradient on a plane through the middle of the transwell for NF after 72 h. **(C)** A comparison of the number of exosomes at the membrane for NF and OF groups as a function of time.

To mimic the experimental setup depicted in Figure 1, a 3-D computational model of the transwell geometry was created, as shown in Figure 4A, and was used for the simulation of exosome transport. This geometry was created using the computer aided geometry (CAD) modeler available in ANSYS (47). The cells that release exosomes were placed at the bottom chamber and the release rates of the exosomes from these cells were taken from the experiments described in the previous section. The experimental measurements described in section Isolation of Exosomes from Conditioned Media and Quantification of Concentration showed that the average exosome release rates were 515.3, and 986.3 per hour per 10^5^ cells, for NF and OF groups, respectively (Figure 1B). In co-culture experiments, internalization of exosomes by immune cells could reduce the exosome concentration near these cells, which could potentially affect the diffusion. However, experimental evidence showed that the concentration change due to internalization was small (Figure 1C) and therefore, exosome internalization measurements from the experiment were assumed negligible and was not modeled. The presented numerical approach will be further refined by adding a sink term in the transport equation (equation 1) to represent exosome internalization in future studies. For the computational simulations, 3.6 × 10^6^ cells were assumed to be placed at the bottom of the transwell for both NF and OF groups, and the exosome source fluxes from the bottom surface were estimated using the exosome release rates from the experiments and the area of the bottom surface of the transwell.

For these simulations, the fluid medium in the transwell arrangement was taken as stagnant. This resulted in the transport of exosomes in the transwell based completely on diffusion. Therefore, the velocity field was set as zero and the Navier-Stokes equations were not used to calculate the flow field. The diffusion coefficient for this simulation was taken as 2.0 × 10^−3^ m^2^/s. The unsteady transport equation was used to estimate the exosome concentration in the computational domain. The initial concentration of the exosomes in the transwell was taken as zero and the computational simulation calculated the time evolution of exosome concentration in the transwell. Simulations were carried out for 72 h and the concentration of exosomes in the computational domain for NF and the comparison of the number of exosomes that reached the membrane for both NF and OF groups are shown in Figures 4B,C.

### Modeling the Tumor Microenvironment

**Figure 5:**
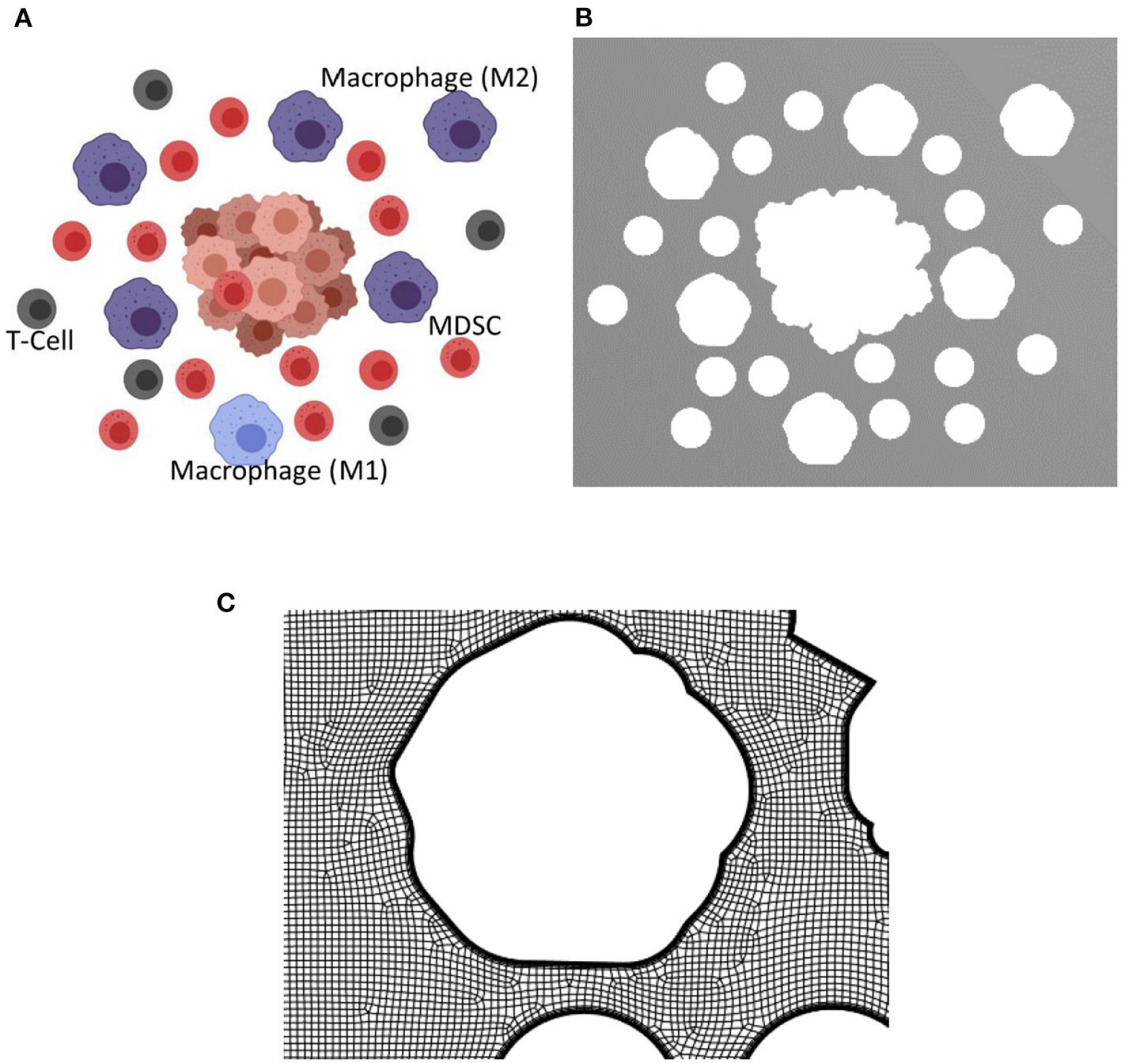
Biorender image, computational domain, and a view of the mesh for the tumor microenvironment with macrophages, T cells, and MDSCs. The tumor microenvironments used for the simulations are created using Biorendor (48), and the rendered images is segmented to extract the geometry of various cell types in the computational domain. **(A)** Rendered image using Biorender. **(B)** Extracted computational domain using image processing techniques. **(C)** A zoomed in view of the mesh in TME used for the computational simulation.

**Figure 6:**
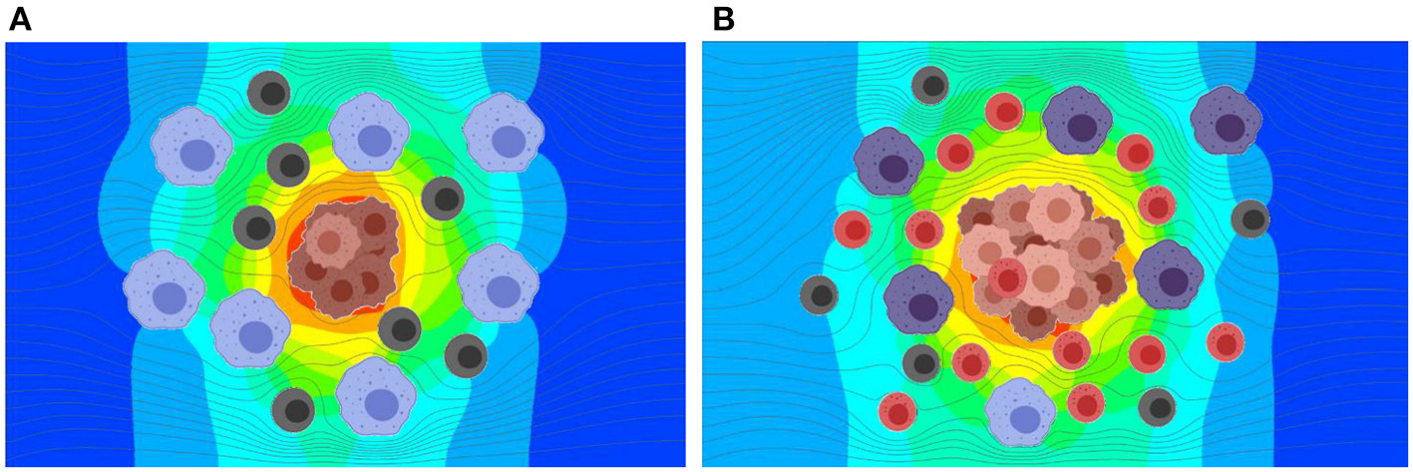
Results from the simulation of interstitial fluid flow and exosome concentration in TME at two stages in tumor growth. Exosomes produced by the tumor cells at a constant rate are carried by the interstitial fluid flow and diffused based on the concentration gradient. **(A)** Predicted exosome concentration in an early stage. **(B)** Predicted exosome concentration in a later stage. The predicted concentration of exosomes is ~3.5 times greater for the later stage as compared to the early stage.

The presence of tumor in a microenvironment not only influences the dynamic behavior of the cells in the microenvironment, but also changes the interstitial fluid flow properties such as pressure and shear stress distributions, and flow patterns. As the tumor grows, obstruction to flow increases and the exosome release rate also increases due to the larger number of cancer cells. To model TME, two different configurations at two different stages of the tumor growth in TMEs with macrophages, T cells, and MDSCs were created using Biorender (48). This approach of generating geometries for TME using Biorender images was considered for this proof-of-principle study. However, more realistic TME from medical images will be considered in follow-up studies. The TME with different cell types at the later stage of growth is shown in Figure 5A. The distribution and proportions of these cell populations were based on what is observed normally in the TME in murine models of both lung and breast cancer (21, 52–55). The extracted geometry using image segmentation techniques and a zoomed-in view of the computational mesh near the tumor surface are shown in Figures 5B,C, respectively. In this simulation, the interstitial fluid was assumed to be coming from the left side of the computational domain with a velocity of 0.75 μm/s. The Navier-Stokes equations together with laminar flow assumptions were used for the calculation of the velocity field in TME. This computed velocity field was used for the evaluation of the advective component of the transport equation. The exosome release rates for the initial and final stages of the tumor were taken as 2.5 and 7.5 exosomes per second, respectively. The computed streamlines in TME and the concentration of exosomes after 45 min are shown in Figure 6.

## Discussion

The validation of the numerical approach for the computational modeling was carried out using the advection and diffusion of a material in a planar shear flow, which has an analytical solution. The order of the discretization of the spatial derivative terms in the transport equation (equation 1) influences the accuracy of computational simulations. To estimate the accuracy of these discretizations and to select an appropriate numerical scheme for the rest of the simulations presented in this paper, simulations were conducted using first order, second order upwind, second order QUICK, and third order MUSCL schemes (43–45). The computed concentrations at 45 min from these simulations were compared with the exact solution (equation 2) in Figure 3. This comparison shows that the results from the second and third order schemes are in very good agreement with the analytical results, and the selected schemes are able to model both advection and diffusion accurately. Therefore, for the rest of the calculations, a second order numerical scheme was used.

The transwell simulations modeled the exosome transport through a stagnant region due to the concentration gradient. As expected, exosome gradient on a plane showed a higher concentration of exosomes at the bottom wall and a lower concentration at the top of both bottom and top wells. In this figure, red represents a higher concentration of exosomes and blue represents a lower concentration. Figure 4C compares the number of exosomes that have reached the membrane at the base of the top well for both NF and OF groups. Computational simulations showed that at all times, a larger number of exosomes reached the membrane for the OF group compared to the NF group. After 72 h, ~2.86 × 10^4^ exosomes reached the membrane for 4T1.2 cells exposed to OF compared 1.58 × 10^4^ exosomes for those exposed to NF. The difference in the number of exosomes passing through the membrane is due to the higher release rate for the OF group, resulting in a higher concentration gradient and faster diffusion. The rate of release of exosomes by the tumor cells in the OF group is ~1.9 times compared to the release rate for the ones in the NF group. This shows the number of exosomes that reached the membrane is close to being linearly proportional to the exosome release rate.

**Figure 7:**
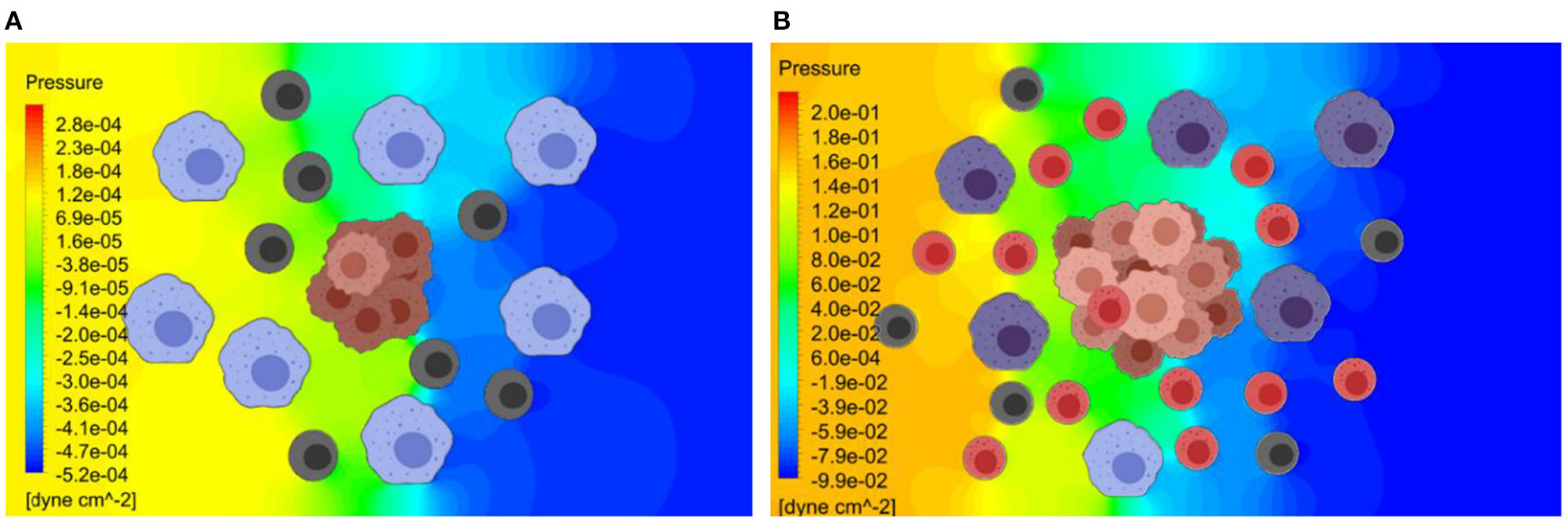
Pressure distribution in the TME at the early and later stages of tumor growth. The Navier-Stokes equations are used to estimate the interstitial fluid flow velocities and the pressure distribution within TME. **(A)** Predicted pressure distribution at early stage. **(B)** Predicted pressure distribution at later stage. The predicted pressure distribution is greater for the later stage of the tumor growth, due to greater obstruction for the flow by a larger tumor.

As shown in Figure 6, the release rate for the later stage of the tumor was taken as a larger value due to the presence of a larger number of cells in the tumor. Here, red represents higher and blue represents lower exosome concentrations. In the later stage (B), the predicted average concentration of the exosomes was ~3.5 times greater than the predicted exosome concentration in the early stage (A). The streamline patterns in the TME for the two different stages of the tumor growth is also plotted in Figure 6. It can be seen from the figure that the flow pattern in the TME is more complex for the later stage of the tumor growth, and this is due to more blockage for the interstitial fluid flow. In addition to the complex flow pattern, the interstitial fluid pressure was higher for the later stages of the tumor growth compared to the early stage, see Figure 7. This could also lead to a higher rate of exosome release due to the larger mechanical forces acting on the tumor surface. However, in this simulation the influence of higher interstitial fluid pressure on exosome release was not taken into account.

The concentration of exosomes in TME is an important parameter in cellular crosstalk and evolution of the dynamic environment. Drug delivery and microRNA delivery via exosomes for specific targets in the TME is an active area of investigation that has significant translational potential. This paper presents a numerical approach to model the transport of exosomes in the TME and quantify exosome concentrations. Results are presented to validate the approach using a benchmark test case, and the accuracy of different numerical schemes are presented by comparing numerical results with the exact solution. The numerical approach presented in our studies is a valuable tool to quantify exosome concentration, exosome gradient, and time evolution of exosome concentration in a TME. This approach can be combined with agent-based models to simulate exosome uptake by immune cells and their polarization. This will enable the evaluation of the influence of different parameters, including the magnitude of force, the frequency and duration of the application, and distance from the tumor cells on the impact of exosomes on immune populations and their polarization rather quickly, without conducting a large number of experiments. This will also make it easier, faster, and more cost-effective to study the effect of the wide range of these parameter values on immune cell polarization that determine the effector or suppressor function at the TME. The use of this numerical approach for *in-vivo* applications requires the proper estimation of tissue properties in a heterogeneous environment, accurate representation of distribution of various cell types in the dynamic ECM environment, and the uptake and polarization of immune cells. However, this approach could serve as a tool to study the propagation of exosomes secreted by the primary tumor lesion and pre-metastatic niche formation in distant tissues, before the cancer cells themselves migrate to the particular tissue, and this will be explored in future studies. Additionally, modeling the dynamics of exosome transport in *in-vitro* models will provide insights to enhance the understanding of exosome transport *in vivo* from the primary tumor site to metastatic sites of the tumor and how exosome transport may influence metastatic niche.

## Data Availability Statement

The original contributions presented in the study are included in the article/supplementary material, further inquiries can be directed to the corresponding author/s.

## Ethics Statement

All cell lines used in the study were screened for mycoplasma and cultured following recommended guidelines.

## Author Contributions

RK, BT, and JB designed and conducted the simulations. KG, YW, SP, JB, and JD designed and conducted the experiments. KG and JB served as the interface to transfer information to and fro between computations and experiments. All authors contributed toward preparation of the manuscript and revisions.

## Conflict of Interest

The authors declare that the research was conducted in the absence of any commercial or financial relationships that could be construed as a potential conflict of interest.

## Abbreviations

CAD: Computer aided geometry
DMEM: Dulbecco's Modified Eagle Medium
ECM: extracellular matrix
EVs: Extracellular vesicles
MDSCs: Myeloid-derive suppressor cells
MUSCL: Monotonic Upstream-centered Scheme for Conservation Laws
NF: No force
OF: Oscillatory force
QUICK: Quadratic Upstream Interpolation for Convective Kinematics
TME: Tumor microenvironment
TNBC: Triple Negative Breast Cancer.
